# Differences in PAR-2 activating potential by king crab (*Paralithodes camtschaticus*), salmon (*Salmo salar*), and bovine (*Bos taurus*) trypsin

**DOI:** 10.1186/1756-0500-6-281

**Published:** 2013-07-20

**Authors:** Anett K Larsen, Kurt Kristiansen, Ingebrigt Sylte, Ole-Morten Seternes, Berit E Bang

**Affiliations:** 1 Department of Occupational- and Environmental Medicine, University Hospital North Norway, Tromsø, Norway; 2 Medical Pharmacology and Toxicology, Department of Medical Biology, Faculty of Health Sciences, University of Tromsø, Tromsø, Norway; 3 Department of Pharmacy, Faculty of Health Sciences, University of Tromsø, Tromsø, Norway

**Keywords:** King crab trypsin, Molecular modelling, Protease-activated receptor-2, Electrostatic interaction

## Abstract

**Background:**

Salmon trypsin is shown to increase secretion of the pro-inflammatory cytokine interleukin (IL)-8 from human airway epithelial cells through activation of PAR-2. Secretion of IL-8 induced by king crab trypsin is observed in a different concentration range compared to salmon trypsin, and seems to be only partially related to PAR-2 activation. This report aim to identify differences in the molecular structure of king crab trypsin (*Paralithodes camtschaticus*) compared to salmon (*Salmo salar*) and bovine trypsin (*Bos taurus*) that might influence the ability to activate protease-activated receptor-2 (PAR-2).

**Results:**

During purification king crab trypsin displayed stronger binding capacity to the anionic column used in fast protein liquid chromatography compared to fish trypsins, and was identified as a slightly bigger molecule. Measurements of enzymatic activity yielded no obvious differences between the trypsins tested. Molecular modelling showed that king crab trypsin has a large area with strong negative electrostatic potential compared to the smaller negative areas in bovine and salmon trypsins. Bovine and salmon trypsins also displayed areas with strong positive electrostatic potential, a feature lacking in the king crab trypsin. Furthermore we have identified 3 divergent positions (Asp^196^, Arg^244^, and Tyr^247^) located near the substrate binding pocket of king crab trypsin that might affect the binding and cleavage of PAR-2.

**Conclusion:**

These preliminary results indicate that electrostatic interactions could be of importance in binding, cleavage and subsequent activation of PAR-2.

## Background

Trypsin is a known activator of protease-activated receptor (PAR)-2 [1,2]. Receptor activation by proteases is achieved by proteolytic cleavage of the N-terminal sequence. This cleavage unmasks a new amino terminus that serves as a tethered ligand that binds to conserved regions in the body of the receptor, resulting in the initiation of signal transduction [3]. Exogenously applied synthetic peptides based on the sequence of the tethered ligand are also capable of activating PARs by directly binding to the body of the receptor. To date, four PARs have been cloned and characterized; PAR-1, PAR-2, PAR-3, and PAR-4. PARs have emerged as important receptors in airway inflammation and allergy, and PARs are expressed in all cell types that participate in the inflammatory response of the lung; epithelial cells, mast cells, macrophages, infiltrated neutrophils and eosinophils, fibroblasts, smooth muscle cells, endothelial cells, lymphocytes, and neurons [4,5].

Our previous work has confirmed that purified salmon trypsin increase secretion of the pro-inflammatory cytokine interleukin (IL)-8 from human airway epithelial cells through activation of PAR-2 [6]. Secretion of cytokines from the airway epithelium contributes to an inflammation response and can be induced by both endogenous and exogenous proteases [3]. Based on the knowledge that occupational airway symptoms are frequently presented by workers handling different species of fish and crustaceans [7-15], we have tested several types of seafood trypsins in our cell based assays. This in order to investigate possible initiation of signal transduction connected to inflammation processes in human airway epithelial cells [6,16]. During purification of numerous fish trypsins (Atlantic salmon [*Salmo salar*], sardine [*Sardinops melanostictus*], anchovy [*Engraulis japonicus*], jacopever [*Sebastes schlegelii*], yellow tail [*Seriola quinqueradiata*], spotted mackerel [*Scomber australasicus*] ) and trypsin from the king crab (*Paralithodes camtschaticus*) by fast protein liquid chromatography (FPLC), we observed that king crab trypsin bound stronger to the anionic column compared to the fish trypsins we purified.

Molecular size, conformation and electrostatic potential will influence on a molecule’s ability to bind and interact with signalling partners. To bind tightly, the ligand must possess a shape and a charge distribution that are complementary to the target receptor [17,18]. In the molecular complex formed, attractive van der Waals and electrostatic (charge-charge) interactions are made across the binding interface. While the van der Waals interactions are relatively non-specific and small in magnitude, the electrostatic (charge-charge) interactions are highly specific and act over a significantly longer range. A small chemical change as conversion of one amino acid from L- to D- form or substitution of amino acids can inactivate the molecule, as the receptor may fail to bind the altered form or bind it less efficiently. The nature of the interaction between two signalling partners will influence upon downstream signalling pathways following molecular interaction leading to activation and transmission of the molecular signal (full or partial agonists) or inactivation without signal transduction (antagonists) [19].

We decided to explore the observed divergence between king crab and fish trypsins further. Purified salmon, sardine, bovine and king crab trypsins were evaluated by their ability to hydrolyze a chromogenic substrate (DL-BAPNA) and molecular modelling was executed of salmon, bovine and king crab trypsins for comparison of molecular structure and possibly identification of important amino acids in the trypsin – PAR-2 interaction.

## Methods

### Materials

Na-Benzoyl-_D,L_-arginine 4-nitroanilide hydrochloride (DL-BAPNA) and trypsin from bovine pancreas (T7309) were purchased from Sigma-Aldrich, MO, USA. Purified salmon trypsin was kindly provided by Dr. Nils Peder Willassen and Dr. Ronny Helland (University of Tromsø). King crab trypsin was manufactured by Dr. Galina N. Rudenskaya (Moscow State University), and the sardine trypsin was supplemented by Dr. Hideki Kishimura (Hokkaido University).

### Protein determination

The protein concentration was determined with a NanoDrop ND-1000 spectrophotometer (Thermo Scientific) using the Protein A280 determination module.

### Fast protein liquid chromatography (FPLC)

All purification steps were carried out at 0-4°C. The freeze dried trypsins prepared according to Rudenskaya *et al*. [20], Kislitsyn *et al*. [21], and Kishimura *et al*. [22] were re-suspended in 25 mM TrisHCl, pH 7.5 and applied to a 1.5 ml benzamidine-sepharose 6B column equilibrated with 25 mM TrisHCl, 10 mM CaCl_2_, 500 mM NaCl, pH 7.5. Bound trypsin was eluted using 120 mM benzamidine and collected in 1.5 ml fractions. All fractions with enzymatic activity measured by the serine protease assay (DL-BAPNA) were pooled and dialyzed against 25 mM TrisHCl, 10 mM CaCl_2_, pH 7.5 at 4°C over night using 10K Slide-A-Lyzer dialysis cassettes from Pierce, IL, USA. The following day the benzamidine purified trypsins were applied to a 1 ml Resource Q ion exchange column equilibrated with 25 mM TrisHCl, 10 mM CaCl_2_, pH 7.5 and the enzymes were eluted with 1 M NaCl using a 7.5% gradient for 10 fractions (total of 5 ml) followed by a linear gradient rising to 100% in 20 fractions (total of 10 ml). Fractions corresponding to the observed peaks were tested for enzymatic activity and pooled before dialysis as described previously.

### SDS-PAGE

After purification the trypsins were run on a sodium dodecyl sulfate polyacrylamide gel electrophoresis (SDS-PAGE) (4-12% NUPAGE; Invitrogen) and stained with SilverQuest^TM^ Silver Staining Kit (Invitrogen) for verification of their purity.

### Protease activity determination

The enzymatic activity of the purified trypsins was determined by a serine protease assay where the hydrolyzation of a chromogenic substrate (DL-BAPNA) was measured spectrophotochemically by the increase in absorbance at 405 nm at room temperature or 37°C for the length of 10 min. The substrate was diluted in substrate buffer (25 mM TrisHCl, 10 mM CaCl_2_, 2% (v/v) DMSO, pH 8.1) and used at a final concentration of 0.5 mM. The activity was measured in a total volume of 250 μl (10 μl of enzyme and 240 μl of diluted substrate) in clear, 96 well trays with flat bottom (BD Falcon, NJ, USA). The results were expressed as units (U)/ml [23], and one unit of activity was defined as 1 μmol substrate hydrolyzed per minute using an extinction coefficient of 8800 M^-1^cm^-1^[24]. The calculations were made using the following formula: Unit : dA/dt × 1/(ϵ × optical path length × 10^6^) × V_final_, where dA/dt = rate of absorbance change and ϵ = extinction coefficient.

### Molecular modelling

A homology model of king crab trypsin (*Paralithodes camtschaticus*; TREMBL accession code: Q8WR10_PARCM) was built by using ICM3.5 [25] and the crystal structure of Atlantic salmon trypsin (pdb code: 1hj8) as a structural template. The homology model of king crab trypsin, the structures of Atlantic salmon trypsin (pdb code: 1hj8) and bovine trypsin (pdb code: 1s0r) were superimposed on the thrombin-PAR-4 structure (pdb code: 2pv9; [26]). The backbone of the catalytic site residues in the trypsins were superimposed with the corresponding residues of the PAR-4-thrombin complex. A model of the N-terminal fragment of human PAR-2 (Gly^28^-Val^53^) was build and superimposed on top of the corresponding PAR-4 segment in the crystal structure of the thrombin-PAR-4 complex. Electrostatic potentials were calculated on the molecular surfaces of the model and structures of the trypsins.

## Results

### SDS-PAGE

**Figure 1:**
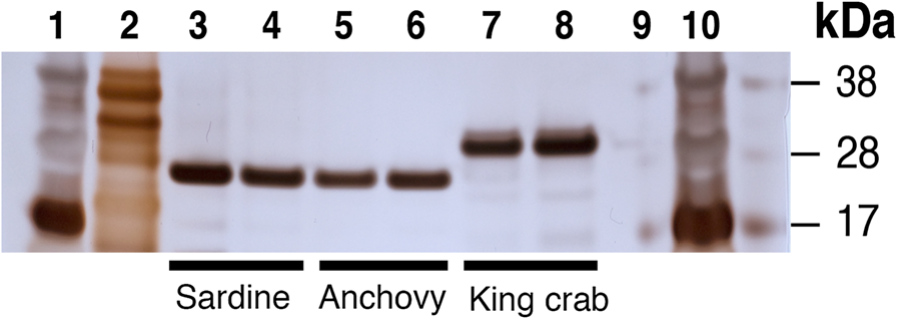
**Purified king crab trypsin differs in size from purified sardine trypsin.** 250 ng of purified king crab and sardine trypsin were run on a SDS-PAGE gel and stained with silver staining for detection. The result shows that purified king crab trypsin (lane 7 and 8) is a slightly bigger molecule residing in the 28 – 30 kDa area compared to the purified sardine trypsin (lane 3 and 4) at 24 – 25 kDa. Additional purified fish trypsins tested (anchovy (lane 5 and 6), yellow tail, jacopever, spotted mackerel (not shown)) displayed similar size as purified sardine trypsin. Lane 1, 2 and 10 contain protein standards, respectively 10 μl SeeBlue®,10 μl Mark 12™, 16 μl SeeBlue®. Lane 9 is empty.

King crab trypsin is reported to be in the size range of 23 – 29 kDa (Kislitsyn *et al*., [21]; Rudenskaya *et al*., [20]). Our results from SDS-PAGE reveal a protein in the 28 – 29 kDa range (Figure 1) compared to sardine trypsin at 24 kDa [22] (Figure 1), and salmon trypsin at 23, 7 – 25 kDa [23,27].

### Effect of temperature on enzymatic activity

**Figure 2:**
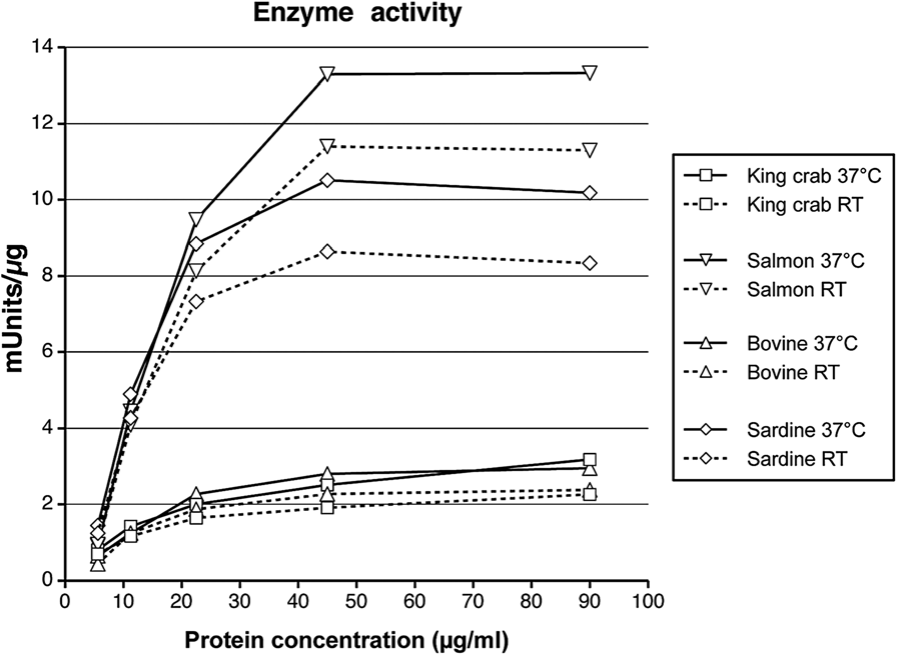
**Protease activity.** The enzyme activity of purified king crab, sardine, salmon, and bovine trypsin was determined by a serine protease assay conducted at room temperature (RT) and 37°C. The protease activity was measured in solutions with increasing protein concentration and results given as mUnits (mU)/μg protein. Total volume of the reaction was 250 μl consisting of 10 μl of purified enzyme and 240 μl of substrate (DL-BAPNA) diluted in substrate buffer. For the measurements at 37°C the substrate buffer was incubated at 37°C for the appropriate time prior to use and the temperature adjusted to 37°C in the chamber of the spectrophotometer.

The effect of temperature on the enzymatic activity of four different trypsins (bovine, salmon, sardine, and king crab) was examined using the substrate DL-BAPNA at room temperature and 37°C. As depicted in Figure 2, the total enzymatic activity of all trypsins decreased with lowered protein concentration. Comparing the enzyme activities at room temperature showed that salmon and sardine trypsin exhibited higher enzyme activity (11.3 and 8.34 mU/μg, respectively), as compared to king crab and bovine trypsin (2.26 and 2.38 mU/μg), using enzyme solutions with a protein concentration of 90 μg/ml. All trypsins showed an increased enzymatic activity when increasing the temperature to 37°C (Figure 2). The purified king crab trypsin showed the most pronounced increment with a 29% rise in enzymatic activity at the highest protein concentration, compared to 19.3% in the bovine trypsin, 18.1% in the purified sardine trypsin, and 15.2% in the purified salmon trypsin. The mean rise in enzymatic activity was 20.6% for the purified king crab trypsin, 17.7% for the bovine trypsin, 15.9% for the purified sardine trypsin, and 9.6% for the purified salmon trypsin. As for the total enzymatic activity, the degree of increment was also reduced with lowered protein concentration for all trypsins.

### Molecular modelling shows differences in the protein structure of the king crab trypsin compared to salmon and bovine trypsin

**Figure 3:**
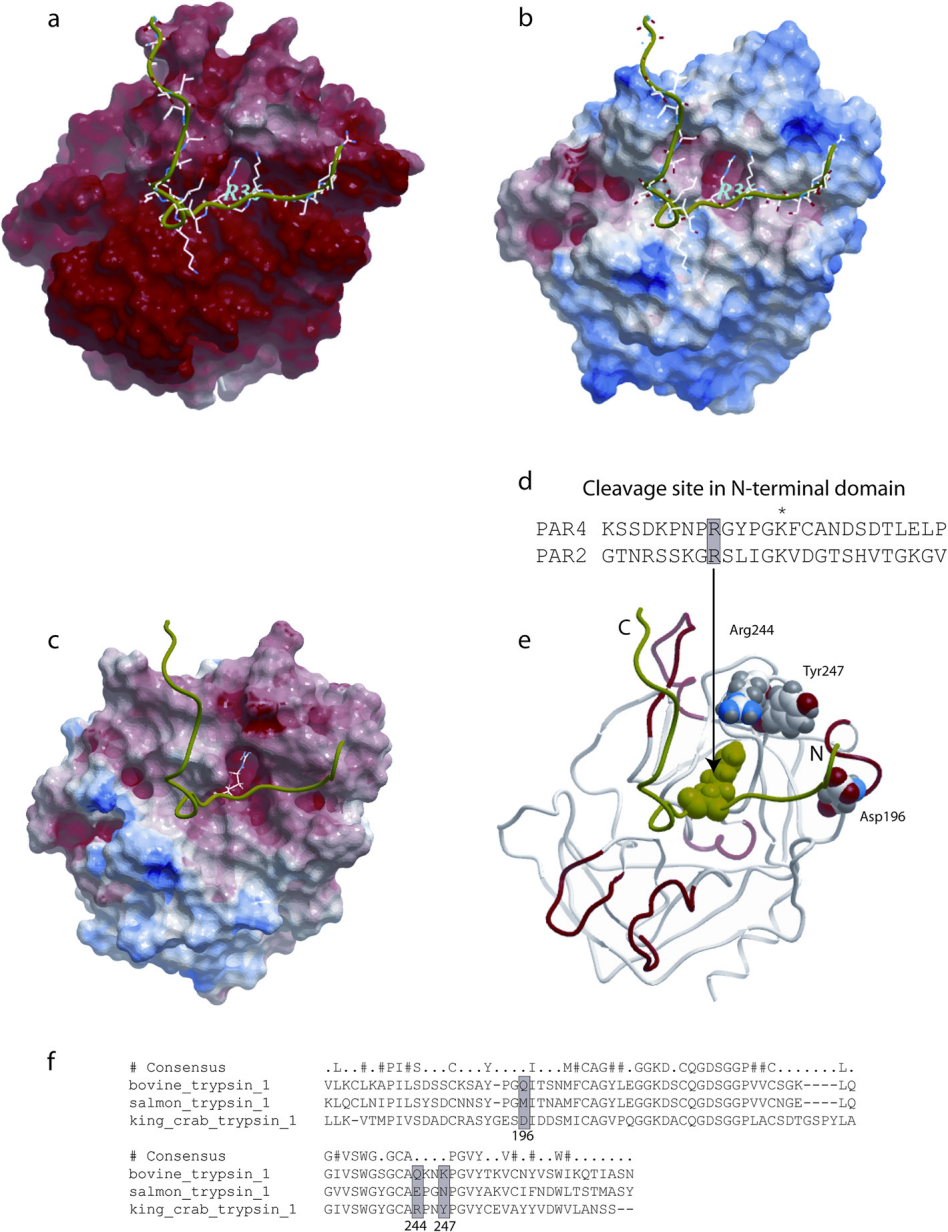
**Molecular modelling of king crab**, **salmon**, **and bovine trypsin. a** – **c**: Electrostatic surfaces of (A) king crab trypsin (homology model), (B) bovine trypsin (pdb code: 1s0r), and (C) Atlantic salmon trypsin (pdb code: 1hj8). The most electropositive potential is shown in blue, whereas the most electronegative potential is shown in red. The PAR-2 agonist peptide is displayed in green ribbon. **d**: The N-terminal fragment of PAR-4 and PAR-2, Arg at the cleavage site (shaded), *: a conserved positive charged residue in tethered peptides. **e** and **f**: The location of 3 binding site residues in the king crab trypsin model that corresponds to different residues in the bovine and Atlantic salmon trypsin (shaded boxes in the structural alignment). Asp^196^ in king crab trypsin corresponds to Met^175^ in Atlantic salmon and Gln^155^ in bovine trypsin; Arg^244^ in the king crab trypsin corresponds to Glu^221^ in Atlantic salmon and Gln^199^ in bovine trypsin; and Tyr^247^ in king crab trypsin corresponds to Asn^224^ in Atlantic salmon trypsin and to Lys^202^ in bovine trypsin.

The surface of king crab trypsin has a large area with strong negative electrostatic potential (Figure 3a). The surface of bovine trypsin has smaller areas with strong negative potential, in particular around Asp^171^ at the catalytic site, and also areas with strong positive electrostatic potential (Figure 3c).

Comparison of the homology model of king crab trypsin with the structures of Atlantic salmon and bovine trypsin suggested that at least 3 divergent positions are located near the substrate binding pocket (Figure 3d): Asp^196^ in king crab trypsin corresponds to Met^175^ in the Atlantic salmon trypsin structure and to Gln^155^ in the bovine trypsin structure; Arg^244^ in king crab trypsin corresponds to Glu^221^ in Atlantic salmon trypsin and to Gln^199^ in bovine trypsin; while Tyr^247^ in king crab trypsin corresponds to Asn^224^ in salmon trypsin and to Lys^202^ in bovine trypsin.

## Discussion

A recent study by Ramachandran and co-workers [28] reports the ability of PAR-2 to exhibit functional selectivity where the proteolytically revealed tethered ligand (TL) sequence(s) and the mode of its presentation to the receptor (tethered vs. soluble) can confer biased signalling. Thus, PAR-2 can signal to multiple pathways that are differentially triggered by distinct protease-revealed tethered ligands. Differential signalling depending on the activating ligand (termed “agonist-biased signalling” or “functional selectivity”) are also reported for other G protein-coupled receptors [29-32]. The exact factors that lead to activation of different signal pathways during binding of agonist to and cleavage of PAR-2 are unknown. Molecular modelling of the human PAR-1 has revealed various electrostatic, steric, and hydrophobic interactions between receptor and the antagonist used in docking studies [33]. Some residues identified as main points for electrostatic interactions have previously been reported as important in site directed mutagenesis studies for PAR-1 function and activity (Asp^256^ and Glu^347^) [34]. It is tempting to speculate that functional selectivity is a result of different capacity in the agonists to bind at these various interaction points.

Furthermore, the positive Arg^5^ in the agonist peptide is from receptor chimera studies in PAR-1 suggested to interact with the negative Glu^260^ in the second extracellular loop of PAR-1 during receptor activation [35,36]. Since these amino acids are conserved in PAR-2 an Arg^5^-Glu^232^ interaction might operate in recognition of the PAR-2 agonist peptide SLIGRL by the receptor. Changing this residue in the PAR-2 agonist peptide (the positive Arg^5^ in SLIG**R**L to a neutral alanine or a negative glutamatic acid creating SLIG**A**L or SLIG**E**L) markedly reduces the peptides’ potency to cause intracellular Ca^2+^ signalling [37]. The Al-Ani study [37] indicate that changes in the net charge of interacting amino acids influence on activating capacity; a result that may be due to interference with electrostatic interactions.

Differences in IL-8 secreting potential and NF-κB activation have been identified for salmon and king crab trypsin. Both effects are coupled to PAR-2 activation, but only partly for the king crab trypsin [6,16]. Molecular modelling shows that the surface of king crab trypsin has a large area with strong negative electrostatic potential compared to the smaller areas of bovine and salmon trypsins. In addition, these latter trypsins also display areas with strong positive electrostatic potential, a feature lacking in king crab trypsin. Because of the lack of a full amino acid sequence of the sardine trypsin molecule we were not able to do any modelling of and comparison with sardine trypsin.

The modelling of bovine, salmon, and king crab trypsin suggests that at least 3 divergent positions are located near the substrate binding pocket and might affect the binding of substrate to PAR-2:

1) The negative Asp^196^ in king crab trypsin corresponds to the neutral Met^175^ in salmon trypsin and the neutral Gln^155^ in bovine trypsin.

2) The positive Arg^244^ in the king crab trypsin corresponds to the negative Glu^221^ in salmon trypsin and the neutral Gln^199^ in bovine trypsin.

3) The neutral Tyr^247^ in king crab trypsin corresponds to the neutral Asn^224^ in salmon trypsin and to the positive Lys^202^ in bovine trypsin.

It is possible that the positive Arg^36^ and/or positive Lys^34^ of PAR-2 may interact differently with the binding pocket in the three trypsins. Because of differences in the electrostatic potential it is possible that PAR-2 might bind weaker to king crab trypsin than to other trypsins due to repulsive interactions between the positive Lys^34^/ Arg^36^ in PAR-2 and the positively charged Arg^244^ in king crab trypsin. This residue corresponds to a negative amino acid (Glu^221^) in salmon trypsin and a neutral amino acid (Gln^199^) in bovine trypsin. Zhang and co-workers [38] have recently documented that long-range electrostatic interactions presumably play an important role in aligning the PAR-2 N-terminal polypeptide with the activating protease (factor VIIa (FVIIa)) domain during binding and subsequent activation of PAR-2. By molecular simulations they show that positive amino acids in the proximity of the cleavage site of PAR-2 (Arg^31^, Lys^34^, and Arg^36^) are located close to negatively charged residues on the binding pocket surface of FVIIa, whereas the negatively charged Asp^43^ and Glu^56^ are close to positively charged FVIIa residues. Although cell-based control studies were not conclusive (antibodies blocking possible PAR-2 interacting residues in the FVIIa molecule resulted in inhibited tissue factor (TF)-FVIIa signalling through PAR-2, while charge reversal mutations in FVIIa (positive Arg^62^ to negative Glu^62^, and positive Arg^84^ to negative Glu^84^) did not significantly inhibit PAR-2 activation), we can not eliminate the possibility that electrostatic interactions in specific regions guide substrate orientation under physiological conditions. However, the specific FVIIa Arg^62^ – PAR-2 Glu^56^ interaction seems not to be essential for PAR-2 activation by TF-FVIIa.

The measurement of enzymatic activity at different concentrations both at room temperature and 37°C using DL-BAPNA as substrate yielded two separate but similar patterns among the four types of trypsins tested, with bovine and king crab trypsin in the lower area and the fish trypsins in the higher area. During the purification process the bovine trypsin behaved like the fish trypsins with regards to the binding capacity, giving rise to an inconsistency as it had similar enzymatic activity as the king crab trypsin. On the other hand, binding of trypsins to the N-terminal end of PAR-2 for cleavage and subsequent receptor activation might behave different from the binding and cleavage of the substrate in the serine protease assay, giving rise to differences in agonist potential in cell based assays.

These preliminary results indicate that electrostatic interactions can be of importance in binding, cleavage and subsequent activation of PAR-2, and that difference in electrostatic charge in residues at key interacting positions may result in altered potency of the agonist in question. However, more extensive molecular modelling of the entire PAR-2 together with docking studies of different agonists and functional binding assays (Isothermal Titration Calorimetry, Fluorescence Resonance Energy Transfer) to independently quantify the binding capacity of all the trypsins to PAR-2 peptide would be highly interesting. Mutations of key amino acids or generation of chimeric trypsins to investigate the structure and activity relationship, along with cell based assays, are necessary to identify essential residues that might influence upon functional selectivity.

## Conclusion

The results indicate that electrostatic interactions could be of importance in binding, cleavage and subsequent activation of PAR-2 and differences in electrostatic charge in residues at key interacting positions may result in altered potency of the agonists in questions.

### Availability of supporting data

The models constructed in this study are available from the authors upon request.

## Competing interests

The authors declared that they have no competing interest.

## Authors’ contributions

AKL carried out the fast protein liquid chromatography, the SDS-PAGE, the measurements of enzymatic activity and drafted the manuscript. KK carried out the molecular modelling and drafted the appurtenant sections. IS participated in the study design and provided critical evaluation of the manuscript. OMS and BB conceived of the study, participated in its design and coordination and helped to draft the manuscript. All authors read and approved the final manuscript.
